# Investigation of the Role of Mitochondrial DNA in Multiple Sclerosis Susceptibility

**DOI:** 10.1371/journal.pone.0002891

**Published:** 2008-08-06

**Authors:** Maria Ban, Joanna Elson, Amie Walton, Douglas Turnbull, Alastair Compston, Patrick Chinnery, Stephen Sawcer

**Affiliations:** 1 Department of Clinical Neurosciences, University of Cambridge, Addenbrooke's Hospital, Cambridge, United Kingdom; 2 Mitochondrial Research Group, Newcastle University, The Medical School, Framlington Place, Newcastle upon Tyne, United Kingdom; Peninsula Medical School, United Kingdom

## Abstract

Several lines of evidence suggest that mitochondrial genetic factors may influence susceptibility to multiple sclerosis. To explore this hypothesis further, we re-sequenced the mitochondrial genome (mtDNA) from 159 patients with multiple sclerosis and completed a haplogroup analysis including a further 835 patients and 1,506 controls. A trend towards over-representation of super-haplogroup U was the only evidence for association with mtDNA that we identified in these samples. In a parallel analysis of nuclear encoded mitochondrial genes, we also found a trend towards association with the complex I gene, NDUFS2. These results add to the evidence suggesting that variation in mtDNA and nuclear encoded mitochondrial genes may contribute to disease susceptibility in multiple sclerosis.

## Introduction

Multiple sclerosis is a chronic inflammatory disease of the central nervous system in which complex genetic factors exert a profound influence on susceptibility. Although the pathogenesis of the disease is unknown, there is considerable circumstantial evidence implicating the involvement of mitochondria in mechanisms of axonal and glial injury [1], [2]. Accordingly genes that determine the function of these organelles are logical candidates for susceptibility and influences on the clinical course.

Mitochondria are unique amongst cellular organelles for having their own distinct genome, separate from that of nuclear DNA. This mitochondrial genome (mtDNA) consists of a circular double stranded DNA molecule which is 16,569 base pairs in length, and contains a total of 37 genes - 2 ribosomal RNAs, 22 transfer RNAs and 13 polypeptides. These 13 polypeptides are all components of the respiratory chain, the core pathway in oxidative phosphorylation, the activity of which is the primary function of mitochondria. A total of over 80 proteins make up the five complexes in the respiratory chain, the remaining proteins being encoded by genes in the nuclear genome. Since mtDNA is inherited solely from the mother, it may be relevant that evidence for a maternal parent of origin effect has been observed in multiple sclerosis [3].

Perhaps the most compelling clinical evidence indicating a role for mtDNA in determining susceptibility to multiple sclerosis comes from the observation that some patients with mtDNA mutations causing Leber's hereditary optic neuropathy (LHON) go on to develop a demyelinating disease which is clinically and radiologically indistinguishable from multiple sclerosis (apart from the prominence of visual failure) [4]. Evidence for mitochondrial dysfunction in multiple sclerosis outside the context of LHON has also been observed in functional imaging studies [5], pathological analyses [2], [6], [7] and in the animal model of multiple sclerosis, experimental autoimmune encephalomyelitis (EAE) [8].

Encouraged by these observations, several investigators [9]–[13] have sequenced mtDNA from patients with multiple sclerosis in an effort to identify relevant polymorphisms. As relatively few patients were considered in these early studies, it is unsurprising that no multiple sclerosis specific variation emerged. In addition to sequencing mtDNA, selected mitochondrial polymorphisms have also been tested for disease association in case-control cohorts [10], [11], [14]–[20]. Here too the modest number of patients considered (typically <100) has limited the power. The data regarding haplogroups have been somewhat contradictory with some researchers suggesting that haplogroups K and J increase risk [17], [21] while others have found evidence that haplogroup J/T is protective [18]. The possible relevance of LHON related mutations has understandably received attention and a suggestion that secondary LHON mutations (mutations which are more common in LHON but are not directly pathogenic for the condition) increase the risk of multiple sclerosis has been suggested [9], [13], [19], [22] whilst another study showed a decreased risk [13].

Given this body of suggestive evidence implicating the involvement of mitochondria in the pathogenesis of multiple sclerosis, along with the limited power and extent of previous efforts, we reasoned that a more extensive analysis of this promising candidate was required.

## Materials and Methods

### Subjects

All individuals included in this study are from the United Kingdom, self reported as Caucasian and gave informed consent. The study was approved by the Thames Valley Multi-Centre Research Ethics Committee. In total we considered 937 trio families (an affected individual and both parents), 96 additional cases without available parental DNA and 671 unrelated healthy controls (that have already been sequenced for their mtDNA). All affected individuals satisfy Poser criteria [23] for the diagnosis of multiple sclerosis. The demographic features of our affected individuals are unexceptional with an average age of 38.6 years, mean duration of disease of 11.9 years, mean EDSS of 4.3, and gender ratio of approximately 3F∶1M.

### Mitochondrial re-sequencing

Re-sequencing of the mtDNA was carried out using a method previously reported [24]. In summary specific primers were used to sequence, in both the forward and reverse direction, 32 overlapping fragments which between them comprehensively cover the entire mtDNA (primers are listed in Supplementary Table S1). All sequencing reactions were performed using the Applied Biosystems BigDye® Terminator v3.1 cycle sequencing kit according to the manufacturer's recommended conditions. Following the sequencing reaction, excess dye-terminators were removed using AutoScreen-96A Well Plates (GE Healthcare). Electrophoresis was performed on an Applied Biosystems 3700 DNA analyzer and the sequence was compared to the revised Cambridge Reference Sequence (rCRS) [25] using SeqScape software v 2.5 (Applied Biosystems). In the more restricted re-sequencing of the hypervariable segment I (HVSI) region of the D-loop alone, the same method was used but only the D1 and D2 primer-pairs considered.

### Mitochondrial haplogroup typing

For each individual in which the mitochondrial genome was re-sequenced the haplogroup was established based on defined coding region polymorphisms according to Torroni et al [26] (see Table 1). For those individuals that were only sequenced for the HVSI region of the D-loop, haplogroups were classified according to Simoni et al [27] and Richards et al [28] (see Table 2). In these individuals, supplemental typing of the coding 7028, 9055 and 12308 variants was completed to more clearly define the H, U and K groups.

**Table 1:** European haplogroup characterisation based on coding region variants.

Haplogroup	7028	12308	9055	13368	16069
H	**C**	A	G	G	C
J	T	A	G	G	**T**
K	T	**G**	**A**	G	C
T	T	A	G	**A**	C
U	T	**G**	G	G	C

Haplogroups were defined based on the variant present at each of these five nucleotide positions. The nucleotides shown in bold represent the haplogroup defining variant at that nucleotide position.

**Table 2:** European haplogroup characterisation based on HVSI variants.

Haplogroup	HVSI variations (16024–163830)
H	CRS
J	16069, 16126
K	16224, 16311
T	16126, 16294
U1a	189, 249
U1b	249, 327
U2	129C, 189, 362
U3	343
U4	356
U5	270
U7	318T

Haplogroups were defined based on a departure from the revised CRS at the above sites. Typing of the coding region variants 7028, 9055 and 12308 was completed to further confirm the H, U and K haplogroups.

### Nuclear genome genotyping

In total 111 nuclear genome single nucleotide polymorphisms (SNPs) were tested. All SNP genotyping was performed using Applied Biosystems TaqMan methodology according to the manufacturer's recommended conditions. PCR was performed on Applied Biosystems 384 well 9700 Viper PCR machines after which genotypes were called on a 7900 High Throughput Sequence Detection System using SDS software. Where available Applied Biosystems Assay-On-Demand (AoD) products were used (n = 93); for all other variants, we used the Assay-By-Design (AbD) service (n = 18). Primers, basic marker performance measures and single point transmission disequilibrium test (TDT) results as determined using Unphased v3.08 [29] are available for each marker in Supplementary Table S2.

All TaqMan assays were first tested against a panel of 378 samples. No workable assay could be designed for seven (6.3%) variants and a further four (3.6%) were found to be non-polymorphic (see Supplementary Table S2). The remaining 100 assays were typed in a total of 937 trio families. For each assay duplicate typing was attempted in 163 individuals.

### Statistical methods

In the haplogroup analysis, all haplogroups with a frequency of <5% were considered together (resulting in a test with 5 degrees of freedom). Association with individual mitochondrial variants was assessed using Fishers Exact test. In the analysis of the SNPs from the nuclear genome, Mendelian consistency was checked using the Pedcheck program v1.1 [30], while deviation from Hardy-Weinberg equilibrium, genotyping success rate and heterozygosity was determined using the Pedstats program [31]. Transmission Disequilibrium Testing (TDT) was performed using the Unphased program v3.0.8 [29].

## Results

### Mitochondrial genome

To catalogue potentially relevant variation we first re-sequenced the entire mitochondrial genome of 159 patients. In an effort to enrich this initial re-sequencing experiment for mitochondrial variants influencing susceptibility, we included 47 cases with an affected mother. The remaining 112 patients all had at least one affected sibling. By comparison with the rCRS, we identified a total of 635 variants of which 62 were novel (see Supplementary Table S3) in that they do not appear in the online databases (Mitomap http://www.mitomap.org/ and the Human Mitochondrial Genome Databases http://www.genpat.uu.se/mtDB/). These novel variants were identified in 47 cases and included 20 nonsynonymous, 29 synonymous, three tRNA, two rRNA and eight non-coding substitutions. None of these novel variants were seen in more than one individual. Considering the nine major European haplogroups, only five (H, U, K, J and T) had a frequency of greater than 5% in our cohort. No significant difference in the frequency of these common haplogroups was found when comparing our 159 cases with 671 healthy controls (Table 3). Excluding cases and controls with haplogroups other than the common five, left us with a sub-group of individuals effectively matched for haplogroup background consisting of 140 cases and 607 controls. Association with individual variants, present at a frequency of >5% in either the case or control population, was tested within this haplogroup matched subgroup. A total of 74 mtDNA variants were tested and the results for all those showing nominal evidence for association at the 10% significance level are shown in Table 4. Although none of these associations survive Bonferroni correction for multiple testing, we noticed that two of these potentially associated variants are from the HVSI region. Therefore we decided to extend the investigation of this region by re-sequencing the HVSI region in an independent cohort of 860 cases together with their unaffected fathers. Re-sequencing failed in 25 individuals (overall sequencing success rate of 98.5%) so that ultimately we had HVSI data from 835 case-father pairs. In an extension analysis of the 835 case-father pairs plus the original 159 multiple sclerosis cases and 671 controls, we found a trend towards an over-representation of super-haplogroup U (Table 3). Genotyping data from the multiple sclerosis associated HLA-DRB1 gene was available in the majority of cases and fathers. We found no evidence for any significant excess in the frequency of the *1501 allele in individuals carrying super-haplogroup U in either cohort. This independence from *1501 indicates that our observed association is not secondary to any effects of *1501.

**Table 3:** Haplogroup Frequencies.

Haplogroup	Screening Phase^a^		Extension Phase^b^		Patients with optic nerve involvement^c^	
	MS % (n = 159)	Controls % (n = 671)	MS % (n = 994)	Controls % (n = 1506)	MS % (n = 673)	Controls % (n = 1506)
H	44.03	47.24	43.36	44.49	43.98	44.49
J	11.32	14.46	10.16	12.62	8.77	12.62
K	8.18	8.79	10.76	9.56	11.44	9.56
T	9.43	10.13	7.75	10.16	7.58	10.16
U	15.09	9.54	14.29	11.62	14.41	11.62
Other	11.95	9.84	13.68	11.55	13.82	11.55

a p = 0.3450.

b p = 0.0194.

c p = 0.0073.

**Table 4:** Nominally associated variations.

Site	Location in genome	Screening Phase			Extension Phase			Odds Ratio (95% CI)
		Cases MAF % (n = 140)	Controls MAF % (n = 607)	p-value	Cases MAF % (n = 858)	Controls MAF % (n = 1334)	p-value	
16093	D-loop	10.71	3.79	0.0021	8.63	5.40	0.0031	1.65 (1.18–2.32)
263	D-loop	97.14	89.95	0.0043	-	-	-	
5004	ND2^a^	5.00	1.65	0.0257	-	-	-	
16270	D-loop	12.14	6.43	0.0309	10.4	8.02	0.0596	1.33 (0.99–1.78)
13617	ND5^a^	11.43	6.75	0.0757	-	-	-	
3197	16s rRNA	11.43	6.75	0.0758	-	-	-	
9477	COIII^b^	11.43	6.75	0.0758	-	-	-	

Variations in individuals that were not designated to one of the 5 major haplogroups (H, J, T, U and K) were excluded from this analysis.

a Synonymous amino acid change.

b Non-synonymous amino acid change from valine to isoleucine.

We performed a stratified analysis based on phenotype by including only patients with multiple sclerosis that had optic nerve involvement, a phenotype known to be associated with mtDNA variants. No clinical data were available for 119 samples and 202 patients showed no optic nerve involvement. A haplogroup analysis of the remaining 673 patients compared to the controls demonstrated a stronger association (p = 0.007) with an overrepresentation of super-haplogroup U in the patients (Table 3).

None of the potentially associated variants from the HVSI region identified in the screening phase showed any greater evidence for association in our extension analysis (see Table 4). We found no evidence for heterogeneity at these markers across the common haplogroups and thus unsurprisingly found no significant change in the association when considering the common haplogroups as separate strata in a Cochran-Mantel-Haenszel test (results not shown). Three of the potentially associated variants from table 4 that are not contained in the D-loop (13617, 3197 and 9477) were present in the same individuals all belonging to haplogroup U. These three variants, like haplogroup U, were over-represented in multiple sclerosis, indicating that they reflect the same rather than different signals.

### Nuclear encoded mitochondrial genes

In an analysis of mitochondrial dysfunction in multiple sclerosis, Dutta et al [2] identified 23 nuclear encoded mitochondrial genes that were found to have significantly altered expression in multiple sclerosis. For each gene we used the HapMap browser release 20 (http://www.hapmap.org/cgi-perl/gbrowse/hapmap20_B35) to select tagging SNPs that captured all of the common (greater than 5%) variation within that gene. After a validation phase (see methods), 100 nuclear genome SNPs were typed in 937 multiple sclerosis trio families. A detailed list of the SNPs analysed is available in Supplementary Table S2. Six SNPs showed evidence of association with nominal significance (Table 5) and four of these reside in the NDUFS2 gene which is part of Complex I. None of these signals survived even modest Bonferroni correction; nevertheless the four NDUFS2 SNPs are in tight linkage disequilibrium with each other, making it unlikely that these data reflect a genotyping error.

**Table 5:** Markers showing nominally significant TDT results.

Gene	Chromosome	Marker	p-value^a^	Odds Ratio (95% CI)
NDUFS2	1	rs3924264	0.00398	1.23 (1.06–1.43)
NDUFS2	1	rs1136224	0.00865	1.29 (1.06–1.56)
NDUFS2	1	rs3813624	0.0238	1.25 (1.02–1.54)
NDUFS2	1	rs4656994	0.0278	1.19 (1.01–1.40)
ATP5J	21	rs3761326	0.0324	1.22 (1.01–1.47)
ATP5G3	2	rs268230	0.0324	1.21 (1.02–1.42)

a Uncorrected.

## Discussion

The aim of this study was to assess whether mitochondrial genes, both nuclear and mitochondrial encoded, are involved in susceptibility to multiple sclerosis. Haplogroup analysis of the mitochondrial genome identified a trend towards an over-representation of super-haplogroup U. In addition we also found modest evidence of an association with variations in the nuclear gene NDUFS2.

In our analysis of the five most common haplogroups, the finding of a trend towards an over-representation of super-haplogroup U (which encompasses haplogroups U and K) and an under-representation of the J/T haplogroup in cases, is in line with a study of multiple sclerosis cases in the Basque people [18]. Restricting the analysis to those patients with optic nerve involvement strengthened the significance. This finding warrants more detailed assessment in a larger replication cohort of patients in order to distinguish a genuine effect specific to this multiple sclerosis phenotype from a chance finding due to sampling variance. Although our study is one of the largest to have investigated the role of mtDNA haplogroups in multiple sclerosis, it is important to point out that its power is still relatively limited. Even considering a modest 5% significance level, the power curves we have previously described [32] indicate that we were only able to detect (90% or better) common haplogroups conferring a relative risk of >1.6 and that we had almost no power to demonstrate association with any common haplogroups influencing risk by a factor of <1.4. Our data make it unlikely that any common mitochondrial haplogroup exerts a substantial risk but that conclusion does not exclude the possibility of more modest effects, similar to those seen with nuclear genes, whereby risk factors of the order of 1.2 have certainly not been excluded.

In an analysis of individual variants from the mitochondrial genome there were no significant findings. Of the mtDNA variants which showed nominal evidence for association in the screening phase, two in the HVSI region, which were screened in a larger cohort, showed reduced evidence for association. Similar power limitations to those described above apply to our analysis of these other mtDNA variants (and the nuclear variants), and these limitations are intensified in the screening phase where only a modest number of cases (n = 159) was considered.

In this study none of the three primary LHON mutations (at nucleotide position 3460, 11778 and 14484) were carried by any of the patients and we found no evidence for association with any of the secondary LHON mutations (4216, 13708 and 14798). Our results differ to those reported in a recent study in Bulgarian multiple sclerosis patients which showed evidence for an association with the secondary LHON mutation 4216C [19]. In the present study the 4216C allele was present in 24.3% and 27.2% of cases and controls respectively (p = 0.5256), the observed trend being in the opposite direction to that identified in the Bulgarian study [19].

Our analysis of nuclear encoded variants shows a trend suggesting the possible involvement of the NDUFS2 gene from Complex I. This complex is the main mediator of the production of mitochondrial super oxide [33] and is involved in ATP synthesis through the generation of a proton gradient. It is the first and largest subunit involved in the electron transport chain and is comprised of two parts, a hydrophilic catalytic peripheral arm which protrudes into the matrix and is where electron transport occurs and the hydrophobic membrane arm embedded in the mitochondrial matrix and utilised for proton translocation [34]. The flavoproteins and the iron sulphur protein subunits (of which NDUFS2 is one) are hydrophilic, with the remaining nuclear and mitochondrial encoded subunits forming the hydrophobic part. Mutations in the NDUFS2 gene have been shown to result in a decreased activity of Complex I due to defects in the assembly and stability of this complex, and these also affect the stability of Complex III but without reducing its activity [34]. Aside from altered energy production, a disruption in Complex I may also affect the elimination of autoreactive T cells. Mitochondrial encoded proteins are essential for activation induced T cell death (AICD). Specifically Complex I is involved in the generation of reactive oxygen species (ROS) and thus the induction of AICD, an important regulator of autoimmunity, via increased expression of CD95L [35]. Given the role of CD95L in the development of T cells and induction of AICD, mitochondrial damage could result in impaired thymocyte development and the inability to eliminate auto-reactive T cells.

In summary we have completed the largest study to date considering the role of mtDNA and nuclear encoded mitochondrial genes in susceptibility to multiple sclerosis. Our findings provide some support that super-haplogroup U may be a risk factor in multiple sclerosis. In addition we have identified a trend towards an association with the NDUFS2 gene in the Complex I pathway, providing further support that this may be disrupted in multiple sclerosis patients. Further analysis of this gene as well as others in the Complex I pathway is required.

## Supporting Information

Table S1 Primer-pairs used to sequence the entire mtDNA genome.
(0.05 MB DOC)

Table S2 SNP characteristics.
(0.05 MB XLS)

Table S3 Novel Variations.
(0.02 MB DOC)
