# Supplementary material for: Investigation of the Role of Mitochondrial DNA in Multiple Sclerosis Susceptibility
Source: PLoS One. 2008 Aug 6;3(8):e2891. doi: 10.1371/journal.pone.0002891 (PMC2494944; doi:10.1371/journal.pone.0002891)
Supplement: Table S1 — Primer-pairs used to sequence the entire mtDNA genome. (0.05 MB DOC) [file pone.0002891.s001.doc]

Supplementary Table S1 Primer-pairs used to sequence the entire mtDNA genome

| Primer Pair | Forward Primer | Reverse Primer |
| --- | --- | --- |
| Mt1 | TGTAAAACGACGGCCAGTTCACCCTCTAAATCACCAG | CAGGAAACAGCTATGACCGATGGCGGTATATAGGCTGAG |
| Mt2 | TGTAAAACGACGGCCAGTTTAAAACTCAAAGGACCTGGC | CAGGAAACAGCTATGACCCTGGTAGTAAGGTGGAGTGGG |
| Mt3 | TGTAAAACGACGGCCAGTAACTTAACTTGACCGCTCTGAG | CAGGAAACAGCTATGACCATTGGTGGCTGCTTTTAGG |
| Mt4 | TGTAAAACGACGGCCAGTACTGTTAGTCCAAAGAGGAAC | CAGGAAACAGCTATGACCTCGTGGAGCCATTCATACAG |
| Mt5 | TGTAAAACGACGGCCAGTCAGTGACACATGTTTAACGGC | CAGGAAACAGCTATGACCGATTACTCCGGTCTGAACTC |
| Mt6 | TGTAAAACGACGGCCAGTCAGCCGCTATTAAAGGTTCG | CAGGAAACAGCTATGACCGGAGGGGGGTTCATAGTAG |
| Mt7 | TGTAAAACGACGGCCAGTCCTTAGCTCTCACCATCGC | CAGGAAACAGCTATGACCAGAGTGCGTCATATGTTGTTC |
| Mt8 | TGTAAAACGACGGCCAGTAATAAACACCCTCACCACTAC | CAGGAAACAGCTATGACCGTTTATTTCTAGGCCTACTCAG |
| Mt9 | TGTAAAACGACGGCCAGTACACTCATCACAGCGCTAAG | CAGGAAACAGCTATGACCGATTTTGCGTAGCTGGGTTTG |
| Mt10 | TGTAAAACGACGGCCAGTTCCATCATAGCAGGCAGTTG | CAGGAAACAGCTATGACCTGTAGGAGTAGCGTGGTAAGG |
| Mt11 | TGTAAAACGACGGCCAGTACCTCAATCACACTACTCCC | CAGGAAACAGCTATGACCTAGTCAACGGTCGGCGAAC |
| Mt12 | TGTAAAACGACGGCCAGTCACTCAGCCATTTTACCTCAC | CAGGAAACAGCTATGACCATGGCAGGGGGTTTTATATTG |
| Mt13 | TGTAAAACGACGGCCAGTTTAGGGGCCATCAATTTCATC | CAGGAAACAGCTATGACCAAGAAAGATGAATCCTAGGGC |
| Mt14 | TGTAAAACGACGGCCAGTATTTAGCTGACTCGCCACAC | CAGGAAACAGCTATGACCCATCCATATAGTCACTCCAGG |
| Mt15 | TGTAAAACGACGGCCAGTGGCTCATTCATTTCTCTAACAG | CAGGAAACAGCTATGACCGGCAGGATAGTTCAGACGG |
| Mt16 | TGTAAAACGACGGCCAGTTAACATCTCAGACGCTCAGG | CAGGAAACAGCTATGACCTACAGTGGGCTCTAGAGGG |
| Mt17 | TGTAAAACGACGGCCAGTACAGTTTCATGCCCATCGTC | CAGGAAACAGCTATGACCGTATAAGAGATCAGGTTCGTC |
| Mt18 | TGTAAAACGACGGCCAGTACCACCCAACAATGACTAATC | CAGGAAACAGCTATGACCGTTGTCGTGCAGGTAGAGG |
| Mt19 | TGTAAAACGACGGCCAGTATCCTAGAAATCGCTGTCGC | CAGGAAACAGCTATGACCATTAGACTATGGTGAGCTCAG |
| Mt20 | TGTAAAACGACGGCCAGTCATCCGTATTACTCGCATCAG | CAGGAAACAGCTATGACCTAGCCGTTGAGTTGTGGTAG |
| Mt21 | TGTAAAACGACGGCCAGTCAACACCCTCCTAGCCTTAC | CAGGAAACAGCTATGACCAGGCACAATATTGGCTAAGAG |
| Mt22 | TGTAAAACGACGGCCAGTATCGCTCACACCTCATATCC | CAGGAAACAGCTATGACCATGATTAGTTCTGTGGCTGTG |
| Mt23 | TGTAAAACGACGGCCAGTCTAATCTCCCTACAAATCTCC | CAGGAAACAGCTATGACCTAGGTCTGTTTGTCGTAGGC |
| Mt24 | TGTAAAACGACGGCCAGTTCCTTGTACTATCCCTATGAG | CAGGAAACAGCTATGACCCGTGTGAATGAGGGTTTTATG |
| Mt25 | TGTAAAACGACGGCCAGTACAATGGGGCTCACTCACC | CAGGAAACAGCTATGACCGTGGCTCAGTGTCAGTTCG |
| Mt26 | TGTAAAACGACGGCCAGTCATGTGCCTAGACCAAGAAG | CAGGAAACAGCTATGACCCTGATTTGCCTGCTGCTGC |
| Mt27 | TGTAAAACGACGGCCAGTGCCCTTCTAAACGCTAATCC | CAGGAAACAGCTATGACCGGGAGGTTGAAGTGAGAGG |
| Mt28 | TGTAAAACGACGGCCAGTCGGGTCCATCATCCACAAC | CAGGAAACAGCTATGACCGTTAGGTAGTTGAGGTCTAGG |
| Mt29 | TGTAAAACGACGGCCAGTACCTAAAACTCACAGCCCTC | CAGGAAACAGCTATGACCAGGATTGGTGCTGTGGGTG |
| Mt30 | TGTAAAACGACGGCCAGTCAACCACCACCCCATCATAC | CAGGAAACAGCTATGACCAAGGAGTGAGCCGAAGTTTC |
| Mt31 | TGTAAAACGACGGCCAGTATTCATCGACCTCCCCACC | CAGGAAACAGCTATGACCGGTTGTTTGATCCCGTTTCG |
| Mt32 | TGTAAAACGACGGCCAGTAGCCCTAGCAACACTCCAC | CAGGAAACAGCTATGACCTACAAGGACAGGCCCATTTG |
| D1 | TGTAAAACGACGGCCAGTATCGGAGGACAACCAGTAAG | CAGGAAACAGCTATGACCGTGGGTAGGTTTGTTGGTATC |
| D2 | TGTAAAACGACGGCCAGTCTCAACTATCACACATCAACTG | CAGGAAACAGCTATGACCAGATACTGCGACATAGGGTG |
| D3 | TGTAAAACGACGGCCAGTCACCCTATTAACCACTCACG | CAGGAAACAGCTATGACCCTGGTTAGGCTGGTGTTAGG |
| D4 | TGTAAAACGACGGCCAGTGCCACAGCACTTAAACACATC | CAGGAAACAGCTATGACCTGCTGCGTGCTTGATGCTTG |
| Sequencing | TGTAAAACGACGGCCAGT | CAGGAAACAGCTATGACC |
